# Existing evidence on the use of environmental DNA as an operational method for studying rivers: a systematic map and thematic synthesis

**DOI:** 10.1186/s13750-024-00325-6

**Published:** 2024-02-15

**Authors:** R. Cruz-Cano, M. Kolb, R. A. Saldaña-Vázquez, L. Bretón-Deval, N. Cruz-Cano, A. Aldama-Cervantes

**Affiliations:** 1 Institute of Geography, National Autonomous University of Mexico, Circuito Exterior s/n, Ciudad Universitaria, Coyoacán, C.P. 04510 Mexico City, Mexico; 2 Instituto de Investigaciones en Medio Ambiente Xabier Gorostiaga S. J, Universidad Iberoamericana Puebla, San Andrés Cholula, Mexico; 3 Departamento de Microbiología Molecular, Instituto de Biotecnología, National Autonomous University of Mexico, Morelos Cuernavaca, Mexico; 4 Facultad de Estudios Superiores Iztacala, National Autonomous University of Mexico, Avenida de los Barrios s/n, Los Reyes Iztacala, C.P. 54110 Tlalnepantla, Estado de México Mexico; 5 Posgrado en Ciencias del Mar y Limnología, National Autonomous University of Mexico, Circuito Exterior s/n, Ciudad Universitaria, Coyoacán, C.P. 04510 Mexico City, Mexico; 6 Posgrado en Ciencias Biológicas, Universidad Nacional Autónoma de México, Unidad de Posgrado, Edificio D, 1er Piso, Mexico City, Mexico; 7 Consejo Nacional de Ciencia y Tecnología, Avenida de los Insurgentes Sur 1582, Crédito Constructor Benito Juárez, 03940, Ciudad de México, México; 8 Institute of Geophysics, National Autonomous University of Mexico, Circuito Exterior s/n, Ciudad Universitaria, Coyoacán, C.P. 04510 Mexico City, México

**Keywords:** Lotic, Water, Metagenomics, Global trends, ROSES, NGS

## Abstract

**Background:**

Environmental DNA (eDNA) is the DNA that can be extracted from an environmental sample, enabling the monitoring of whole biological communities across a large number of samples, at a potentially lower cost, which can significantly benefit river conservation. A systematic mapping protocol was designed to investigate the use of eDNA in rivers, specifically in terms of research topics, geographic and taxonomic biases, as well as information gaps. Furthermore, the potential research opportunities of eDNA in rivers and possible paths to find this kind of information on available platforms are identified.

**Methods:**

A published systematic map protocol was applied, consisting of a search for published articles and gray literature in two bibliographic databases and one search engine. All search results were submitted to a 2-stage screening for relevance and pertinence in accordance with pre-defined eligibility criteria. Data extraction and codification regarding country of study, year, taxonomic group, sequencing platform, and type of technique employed resulted in a publicly available database.

**Results:**

From 7372 studies initially obtained by the search, 545 met the inclusion criteria spanning a period from 2003 to 2022. The five countries with most studies are: USA (134), Japan (61), China (54), Brazil (29) and the UK (25). The most used fragments to analyze DNA are 16S and COI, whilst 26S and 23S are the least used. Only 84 (15%) of the studies reported hypervariable regions, among which the most used are V4 and V5. Regarding taxonomic groups, fishes are most often studied (176), followed by bacteria (138) and virus (52), while fungi is the least studied group (3). Concerning data availability, 229 (42%) studies provided access to sequencing data.

**Conclusions:**

This study presents a comprehensive analysis of the available evidence regarding the implementation of the eDNA methods in rivers. The findings indicate that since the year 2003, this approach has been applied to aquatic lotic systems, and their recent increase can be attributed to the development of Next-Generation-Sequencing technologies and their reduced costs. However, there is a bias towards high-income countries, particularly USA and Europe. Widespread use and applications of this approach at a global level would allow for the generation of a large amount of information that can be compared between countries to understand if responses of aquatic systems follow similar patterns worldwide.

**Supplementary Information:**

The online version contains supplementary material available at 10.1186/s13750-024-00325-6.

## Background

Biodiversity has significantly declined over the last century, with freshwater populations being the most affected. Between 1970 and 2019, there has been an 81% reduction in freshwater populations [1–4]. It is challenging to develop conservation plans, without knowing which species are present in an ecosystem, so there is a need to improve or develop methods that are more detailed, extensive, fast, and cheap, in order to characterize, identify, and make informed decisions about the future of freshwater bodies and their biodiversity [5, 6]. A fundamental tool for providing data to enable an adequate management of freshwater environments is biological monitoring. This approach obtains information about a particular site and its changes over time, as well as the effects of different impacts on biodiversity and hence, its assessment [2, 7–9]. These types of studies usually are done using conventional methods that involve the identification of species using morphological characters [2, 9, 10]. However, this kind of assessment has some challenging problems due to the phenotypic plasticity, sibling species, different stages of life cycle and seasonality [3, 7, 11]; The lack of experienced taxonomists and properly developed species identification keys are major challenges in this determination method in most countries. Current approaches to surveying conspicuous faunal elements are dependent on suitable field conditions and are limited to the narrow portion of biodiversity that can be found and recorded using the conventional approach. Besides, the morphology-based biodiversity assessment can be invasive, time-consuming, and financially expensive [2, 11, 12].

A method that promises to be effective to improve biodiversity monitoring is the environmental DNA (eDNA) approach, which uses Next-generation sequencing (NGS) tools [2, 13, 14]. NGS refers to the deep, high-throughput, in-parallel DNA sequencing technologies developed a few decades after the Sanger DNA sequencing methods that can provide massive information analysis at much reduced cost (Additional file 1). Genomic studies have rapidly shifted to NGS-based methods, enabling the identification and quantification of multiple species where eDNA has taken advantage of this massive information generation [14–17]. One application of these technologies comes from the discovery that organisms leave traces of their DNA in the environment, and when extracted from environmental samples, is collectively referred to as environmental DNA (eDNA) [2, 18]. It includes a combination of DNA that originates from different sources like feces, saliva, urine and skin cells of animals occupying water bodies and similarly from animals that interact with the environment; these mixtures can consist of DNA from multiple taxa in different life stages, such as vertebrates, invertebrates, bacteria or algae, in sediments, soil, feces or marine and fresh waters. The analysis of eDNA involves the collection of a sample, extraction of the genomic material and the amplification of the DNA. DNA extraction and NGS can be carried out within a few hours, making this technique a fast method for detecting the presence of a target species [2, 10, 15, 18–23].

Environmental DNA metabarcoding, is a method that uses High-Throughput-Sequencing (HTS) to determine the information from a pool of genetic material obtained in a sample, which can be linked to a DNA barcode database, hence the name “metabarcoding” [24]. This approach has emerged as an innovative biodiversity monitoring tool that enables the rapid classification of multiple taxa without the need of a taxonomist or local knowledge [19, 21, 25]. Another cost-effective, fast and non-invasive eDNA technique is Whole Genome Sequencing (WGS), which enables amplification and sequencing of all genomes of any species in an environmental sample, and transcriptomics, where all the expressed and transcribed genes present in an environmental sample can be characterized [2, 21].

eDNA approaches have been used mainly in marine environments and lentic systems, due to their stability and predictability, thus, enabling easy sampling protocols [2, 6, 22, 23, 25–30]. However, many other types of water samples have been collected from a variety of water bodies including laboratory tanks, artificial and natural ponds or lakes, lagoons, streams and rivers [6, 11, 22, 23, 26, 31]. Many projects and consortia for sequencing metagenomes have been launched in the past 10 years, such as the TerraGenome project for soils and the Tara Oceans project on the microbiome, eukaryotic plankton, and viromes of the global oceans [16]. Despite rivers playing a crucial role in biodiversity conservation, human use, and maintaining ecosystems´ regulation processes by transporting water and nutrients to almost all zones on the planet and draining nearly 75% of terrestrial surface [1, 4, 32–34], the information about the research in lotic water bodies through an eDNA perspective remains scarce.

The formulation of the present research question and scoping of the systematic map was discussed with different Mexican research institutions from different perspectives (ecological, biological, biotechnology, marine sciences and limnology) and a Non-Governmental Organization specialized in citizen science (Global Water Watch Mexico). Our aim is to improve the knowledge of this approach as well as its application in a global context to understand the pertinence of implementing the study of rivers via eDNA to produce data relevant to stakeholders and decision-makers.

## Objective of the review

The objective of this systematic map is to identify, map and describe the evidence of eDNA research in rivers. This study analyzes the application of the eDNA approach in rivers, identifies geographic and taxonomic biases, as well as information gaps and future research opportunities.

### Primary question

What evidence exists on the use of environmental DNA as an operational method for studying rivers?

### Secondary questions

What are the spatiotemporal trends of eDNA studies in rivers?

Which taxonomic groups have been studied with this approach?

What are the most used methods for eDNA studies of rivers?

Which are the most used sequencing platforms for eDNA studies of rivers?

Which are the most used conserved regions and variable regions in eDNA studies of rivers?

How much of the data generated in the studies is available for the public?

The question components are defined as follows:

- Population: Lotic water bodies (continuous movement of water) particularly rivers; except those related to estuaries or with influence of brackish water.
- Intervention: Use of eDNA (DNA obtained from an environmental matrix).
- Outcome: All outcomes related to the studied population, including data about taxonomic groups studied, sequencing platforms, environmental matrix used (water, sediment, biofilm, mixed sample), biodiversity, community structure, detected pathogens, type of technique applied (amplicon, WGS, transcriptomic), conserved sequences employed (if applicable), and public availability of sequencing data.

## Methods

The strategy designed for this systematic map was developed in accordance with the Guidelines and Standards for Evidence Synthesis in Environmental Management [35]. The ROSES (Reporting Standards for Systematic Evidence Syntheses in environmental research) format for Reporting Standards for Systematic Map Protocols [36, 37] was implemented, as well as the procedures explained in the protocol [38] previously published to this systematic map.

### Deviations of the protocol

The systematic map was modified from a previous protocol [38] as follows (Additional files 2, 3):

- Prior to the application of the systematic map protocol, a bibliometric analysis was performed to have a general picture of eDNA use, as well as the main keywords used, and their relationships. This bibliometric analysis database was generated with the platform Dimensions and helped us to delimitate the words to be used in the search strings [39] (Additional file 4).
- A new review question was included: “Which are the most used conserved regions and variable regions in eDNA studies of rivers?
- A complementary thematic synthesis was developed, as the combination of systematic mapping and thematic synthesis is most appropriate to analyze the available qualitative evidence [40, 41].

### Search for articles

#### Search terms and strings

During the search, the field "topic" that includes title, abstract and keywords has been used and searches considered full text. The scoping search string used the Web of Science format, considering only English-language studies, using Booleans (AND, OR) and wildcards (Additional file 5). As the main objective of this research is focused on environmental DNA applied to riverine systems, relevant terms do not include terms like brackish aquatic systems, lakes or swamps. There were no new relevant search terms during the extraction process. The final search string was adapted in concordance to the format of the database or web-based engine, and the search was conducted on title, abstract and keywords (Additional file 5).

#### Search sources

A search in two bibliographic databases was conducted (Web of Science and SCOPUS). also, the search engine Google Scholar was used to identify additional gray literature [35]. Only the first 100 results shown by the search engine were considered for screening. The search was conducted using institutional subscriptions and licenses (Universidad Nacional Autónoma de México digital library and digital database (comprised publications since 1900), with an initial search in January 2022, and its update in August 2022.

#### Comprehensiveness of the search

The comprehensiveness of our search string was tested using 10 papers (selected independently from the search) considered as relevant by the whole team as an indicator of a successful search (Additional file 6). After several adjustments, the final search string captured 9 of 10 from those key papers (Additional file 6). Additionally, the selected search terms were discussed and agreed to by all the team members in order to ensure a comprehensive search.

### Article screening and selection criteria

#### Screening process

Deduplication of search result was done in Excel (rearrange function and manually removed any repeated documents with the same title). To ensure consistency and accuracy of the review, the team underwent previous training. Our screening strategy consisted of two stages: (1) First the title and abstract were reviewed based on a decision tree in concordance with the study objective that was designed by all the review team (Additional file 7). Each study was screened by all members, and any study that was deemed as “uncertain” about inclusion/exclusion proceeded to stage two of the screening process for a full text screening. (2) The second stage consisted in the full text screening of those studies that passed the first stage, as well as those deemed as “uncertain”. The selected articles were uploaded to a citation manager (Mendeley 2022) and displayed in an Excel datasheet (Additional file 8) in a shared folder where RICC, AAC and NBCC thoroughly screened the articles for relevant data. Additionally, excluded studies as well as their reasons for the exclusion were listed in an Excel datasheet and provided as Additional file 8.

### Eligibility criteria

Articles were selected according to inclusion/exclusion criteria during screening stage 2 (Table 1). The criteria were applied as described in the review protocol without deviations. The main criteria were related to study type, language, and the PIO framework (Population-Intervention-Outcome) as suggested in the PROCEED platform. There were no restrictions about the country in which the studies were developed, nor for the year of publication.

**Table 1:** Inclusion and Exclusion criteria employed in the present systematic mapping

	Inclusion criteria	Exclusion criteria
Type of study	Original articles, studies presented in theses and conferences	Books, chapters, letters to editor, review studies (systematic reviews, meta-analysis), modelling studies that didn´t take environmental samples
Language	English	Non-English papers
Population	Lotic water bodies (rivers and some of its synonyms as: stream, watershed, catchment, basin, watercourse, waterway, brook, tributary, channel, creek, etc.) where the use of environmental DNA was applied	Wastewater Treatment Plants, sewage, lakes, microcosm experiments, estuarine or marine systems
Intervention/exposure	Use of environmental DNA (eDNA) framework/technique for studying rivers	There was no use of environmental DNA framework/technique
Outcome	Report of eDNA persistence, distribution, comparison of techniques where eDNA is used, characterization of community composition or the presence/absence of some species via eDNA use	No report of eDNA persistence, distribution, comparison of techniques where eDNA is used, characterization of community composition or the presence/absence of some species via eDNA use. Studies in which there was only a Draft Genome Complete Genome Sequencing Isolated
Study design	Experimental studies that included sampling of eDNA through some environmental matrix as: water, sediment or biofilm	–
	Study designs that used eDNA for modelling persistence, resistance or distribution of eDNA	
	Studies that used eDNA for detection of species focusing in monitoring species at risk, exotic/invasive species, or those which have an important role on human health	
	Studies that compare the use of eDNA versus conventional techniques for monitoring and identify organisms	
	Studies that focus on determine a baseline for biodiversity of a lotic water body	
Geography	There was no limitation for geographic areas	–
Period	There was no time limit for studies	–

### Consistency checking

The screening was performed by three reviewers (RICC, AAC and NBCC) and included a consistency check of 10% randomly selected articles (50 studies) that were screened by the two other members of the review team to confirm the consistency of article selection. All discrepancies about screened articles were discussed by the 3 members of the reviewing team. Consistency of screening was measured based on two outcomes about its inclusion: “Yes” or “No”. There was a high Kappa coefficient (k) after repeating the process two times (k = 0.83, z = 1.98, p-value = 0.0476) (Additional file 9).

### Study validity assessment

We did not undertake a critical appraisal for each study due to the large number of studies encountered in the searches. First, due to the big number of studies that will be encountered in the searches; secondly, because the included articles will be reviewed by all the team in order to identify if there is a study that did not fulfill the criteria; third, because the aim of this study is to describe the location of existent studies and not to analyze the results; and finally, because of the great variability in design, approach, and objectives of the several studies.

### Data coding strategy

After passing the screening stage, each selected study underwent data extraction, which included a consistency check. A random sample of 20 studies was reviewed by two other team members, who used a data sheet in Excel to input meta-data and information about relevant variables. Once a reviewer finished reviewing the articles, 10% of the total studies were analyzed by the other two members of the team to ensure the data extraction was done correctly.

Data extracted included: Bibliographic details (keywords, DOI), study location, intervention, outcome, study design, year of publication, data availability, taxonomic groups (Table 3, Additional file 10). The team also categorized the main topics surveyed from all the documents, in accordance with the criteria described in Tables 2, 3.

**Table 2:** Conceptual categories established according to the study objectives and their description

Topic	Description
Biodiversity assessment (BA)	Studies that assess diversity, composition, distribution, abundance and richness
Antibiotic resistance genes (ARG)	Studies focused in the identification, detection and characterization of bacterial resistance genes to antibiotics through NGS
Functional traits/physiology (PHY)	Focused on determinate functional potential or dominant metabolism-type in a system
Diseases detection (DIS)	Studies focused on detection of virus, and other microorganisms that are considered of medical importance for humans
Monitoring (MON)	Focused on a single species, and its presence/absence in the ecological system and distribution range
Seasonal changes (SEA)	Description of spatiotemporal changes in species composition, population trends, diversity, and their presence
Comparison vs conventional surveys (COMP)	Focused on making a comparison between the use of eDNA methodology vs conventional methodologies, monitoring programs, and identification procedures
Quality assessments (QUA)	Studies that try to determine quality, health or system conditions based on the use of eDNA. This includes effects of pollution on quality of rivers
Exotic/invasive species (EXIV)	Studies focused on identifying through use of eDNA, presence of exotic and/or invasive species that could degrade the system
eDNA degradation/perdurance (DEPE)	Research about eDNA degradation process, rates, persistence and their role on biological studies
Techniques evaluation (TEC)	Evaluation of effectiveness, cost, sample effort, resolution, and generated information of eDNA over other techniques
Community assemblages (COMA)	Focused on characterize a group community (fishes, crustaceans, bacteria, etc.) assemblages
eDNA modelling (MOD)	Models’ construction that try to predict eDNA transport, sites with more eDNA concentration, and other eDNA processes
Species at risk (RIS)	Studies whose objective is to detect, monitor and assess through eDNA, presence, absence, biomass estimation, etcetera of species at risk categories
Bioindicators (IND) Pollution (POL)	Proposal of use some species as bioindicators, based on their eDNA sampling and processing studies that tried to document the pollution effects on a single species or a community level

**Table 3:** Extracted variables, characteristics, and examples

Extracted variable	Description and examples
Year	Year in which the study was published
Country	Country where the study was realized (independently if the authors or the institution that realized the research are from a different country)
Data availability	Public access to sequences runs obtained by environmental DNA (Those data are presented mainly in the form of SRA accession numbers, or bioproject numbers)
Studied group	Taxonomic groups studied or identified through eDNA framework (fishes, plants, fungi, mammals, etc.)
Platform	The sequencing platform used for the genetic material obtained via eDNA (Illumina, 454, Nanopore, etc.)
Technique focus	The technique employed for amplify, assign, and/or process the eDNA sample (metabarcoding, Whole Genome Sequencing, Transcriptomics, etc.)
V region	The hypervariable region reported to be used in the study, for taxonomic resolution of eDNA analysis (V4, V5, V6, etc.)
Fragment	Any of the conserved regions or fragments in case they were reported (16S, 18S, COI, rubcL, ITS, etc.)
Fraction used	The environmental matrix where eDNA was obtained (water, sediment, mixed sample containing water and sediment, etc.)

### Data mapping method

#### Systematic mapping

All the included articles, its coded meta-data and bibliographic information have been available as an Excel document (Additional file 11). This database was processed in R 4.0.2 (R Development Core Team 2020) using packages “ggplot2” and “ggmap” (v3.3.3) [42, 43] for producing figures and tables that allowed us to identify spatiotemporal trends, knowledge gaps and clusters of evidence about the Review Questions.

#### Thematic synthesis

We used a thematic synthesis approach commonly used to synthesize qualitative research, involving the translation of concepts from one study to another through coding, ensuring consistency of interpretation, and using the themes from the outputs to answer a research question [40, 41]. It is considered by some authors as a complementary analysis that takes advantage of the large amount of information generated by systematic mapping, which allows adequately identifying information gaps and biases on particular topics [40, 41]. The process involved coding of text, as well as identify descriptive and analytical themes [40, 41]. Finally, categories for research topics were established in accordance with the extracted variables (Table 2). Those categories were determined considering the focus of the research (methodological comparisons, diseases and public health, assessment of persistence/degradation of eDNA, monitoring of communities, invasive or endangered species, etc.).

## Review findings

**Fig. 1:**
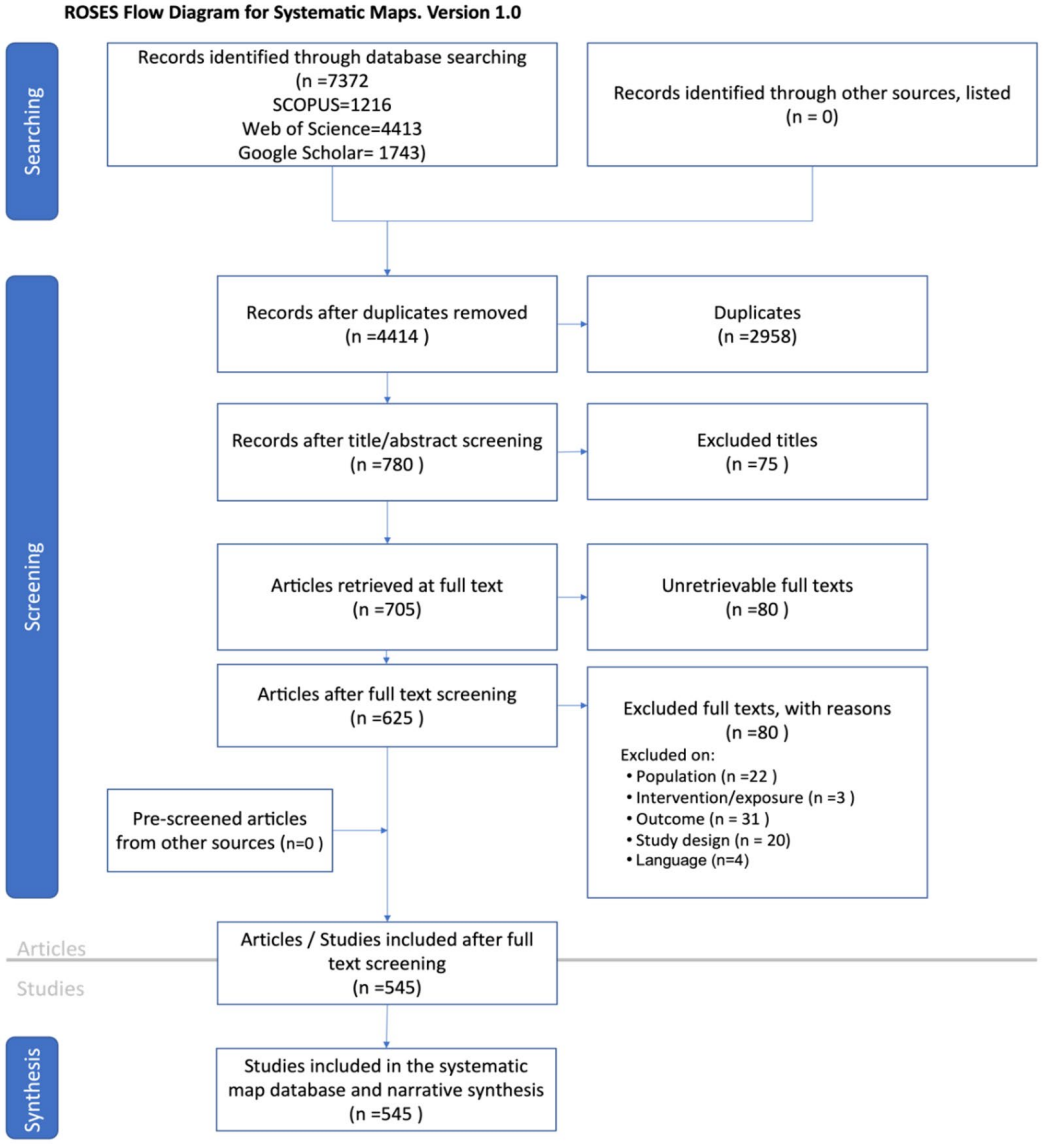
ROSES (Reporting Standards for Systematic Evidence Syntheses in environmental research) flow diagram used in the present study

The search returned 7372 studies that contained all the relevant word combinations in any part of the text. After applying inclusion/exclusion criteria and following the ROSES flow diagram, the number was reduced to 545 (Fig. 1, Additional file 8). It is worth mentioning that the same studies were used for systematic mapping and for thematic synthesis.

### Spatiotemporal trends in publications

**Fig. 2:**
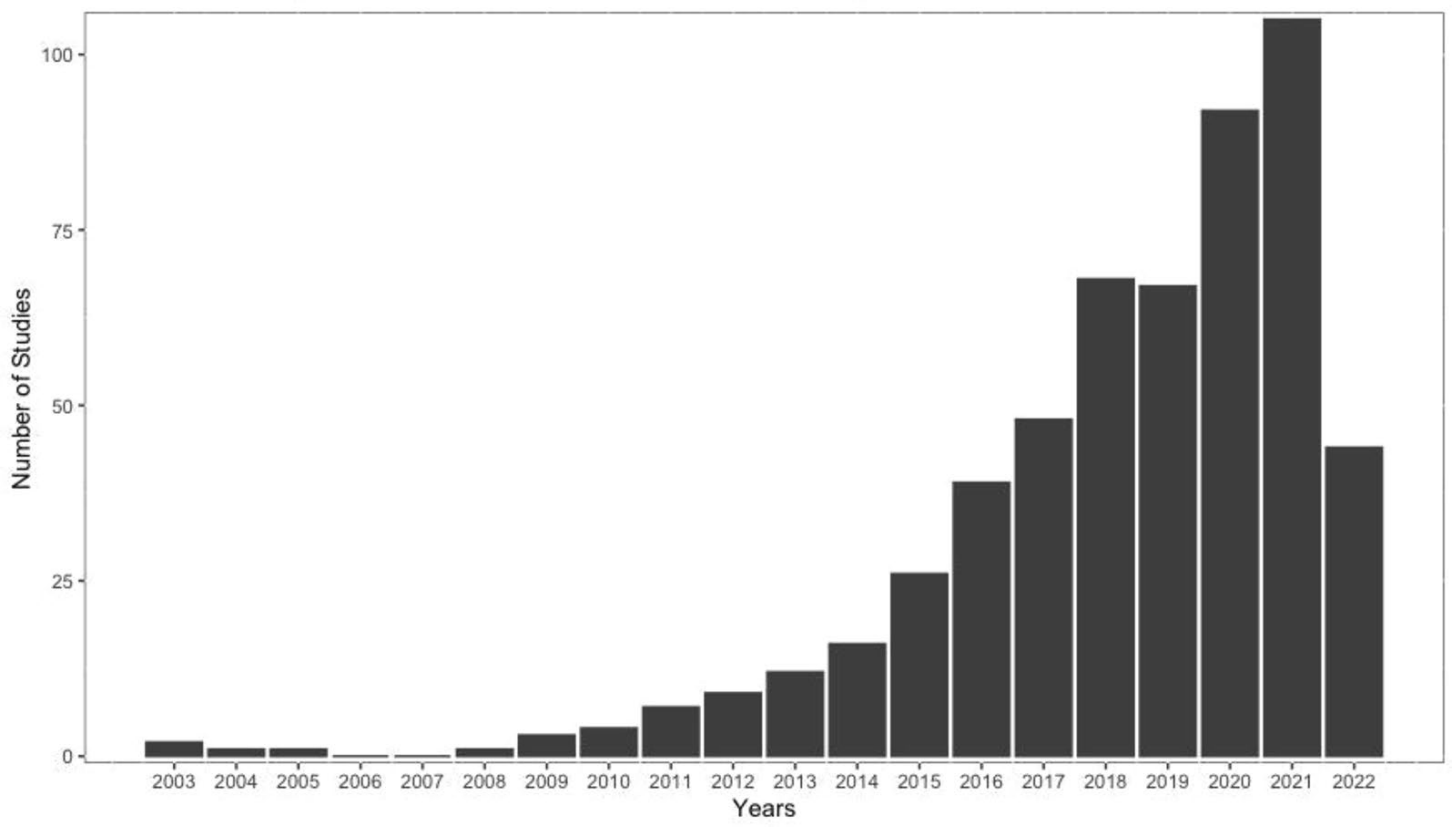
Number of papers that use eDNA in rivers, published from 2003 to August 2022

Over time, there was a significant increase in studies, and in 2021, 105 published studies met all the selection criteria (Fig. 2). It was not until 2008 that publications started mentioning the concept of eDNA and its applications in rivers. Before this year, studies referred to eDNA as “extracellular DNA”, “intracellular DNA” or “bulk DNA”, which were also considered.

**Fig. 3:**
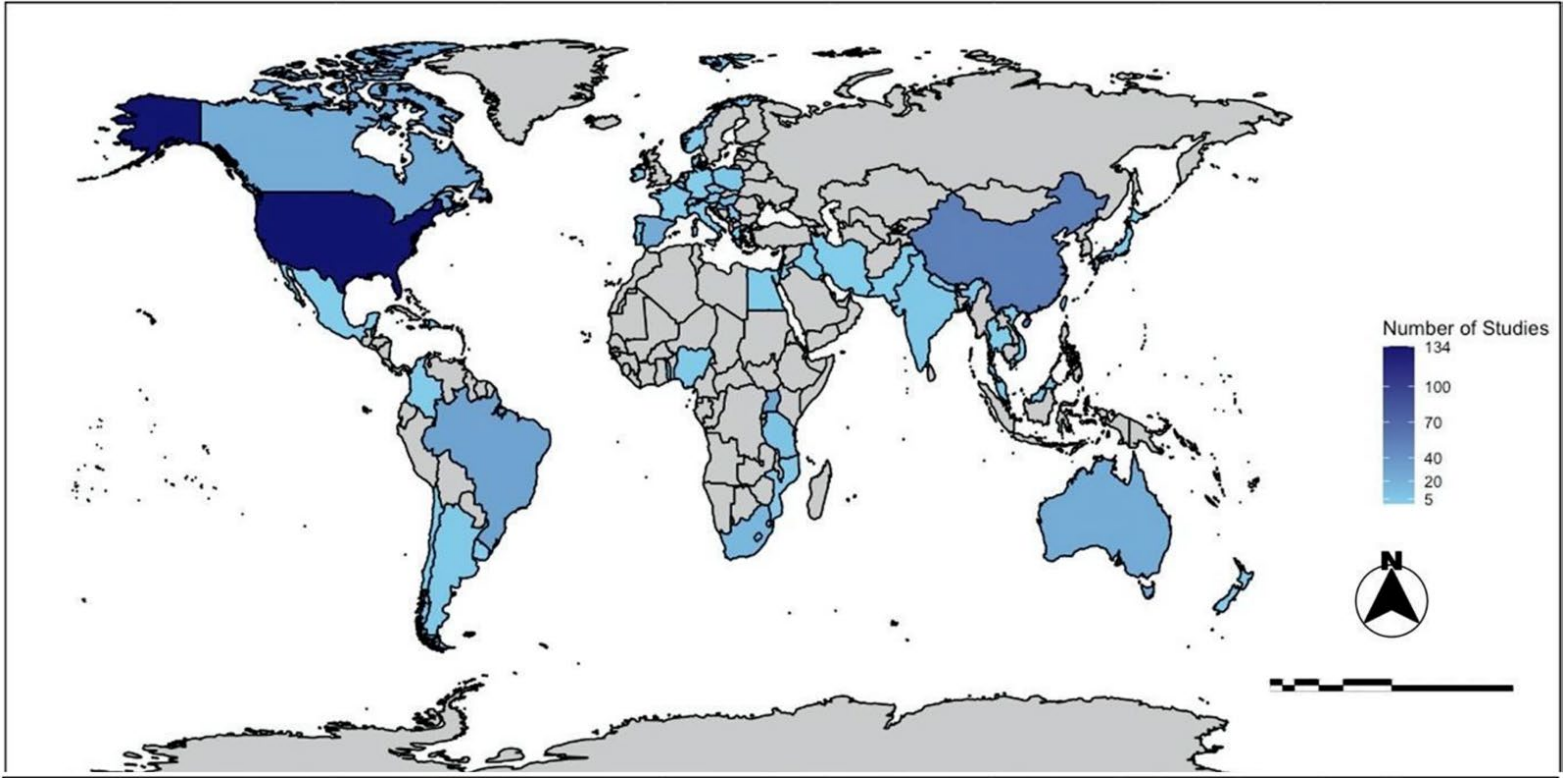
Number of studies using eDNA in rivers per country from 2003 to 2022

Out of the 545 selected studies, the United States of America (USA) had the highest number (134), followed by Japan (61), China (54), Brazil (29), and the United Kingdom (UK) (25). It is important to state that there is a poor coverage of studies in Africa, and the countries that have applied this framework on this continent have just 1 study, except for South Africa (8). Of the 59 countries that have done studies using the eDNA framework, 27 (46%) are members of the OECD (Organization for Economic Co-operation and Development). Although the number of countries that use this framework in Asia and Europe is higher than in other continents, most of the studies (30%) were conducted in two countries on the American continent: USA and Brazil (Fig. 3, Additional file 12).

### Use of conserved fragments hypervariable regions reported

**Fig. 4:**
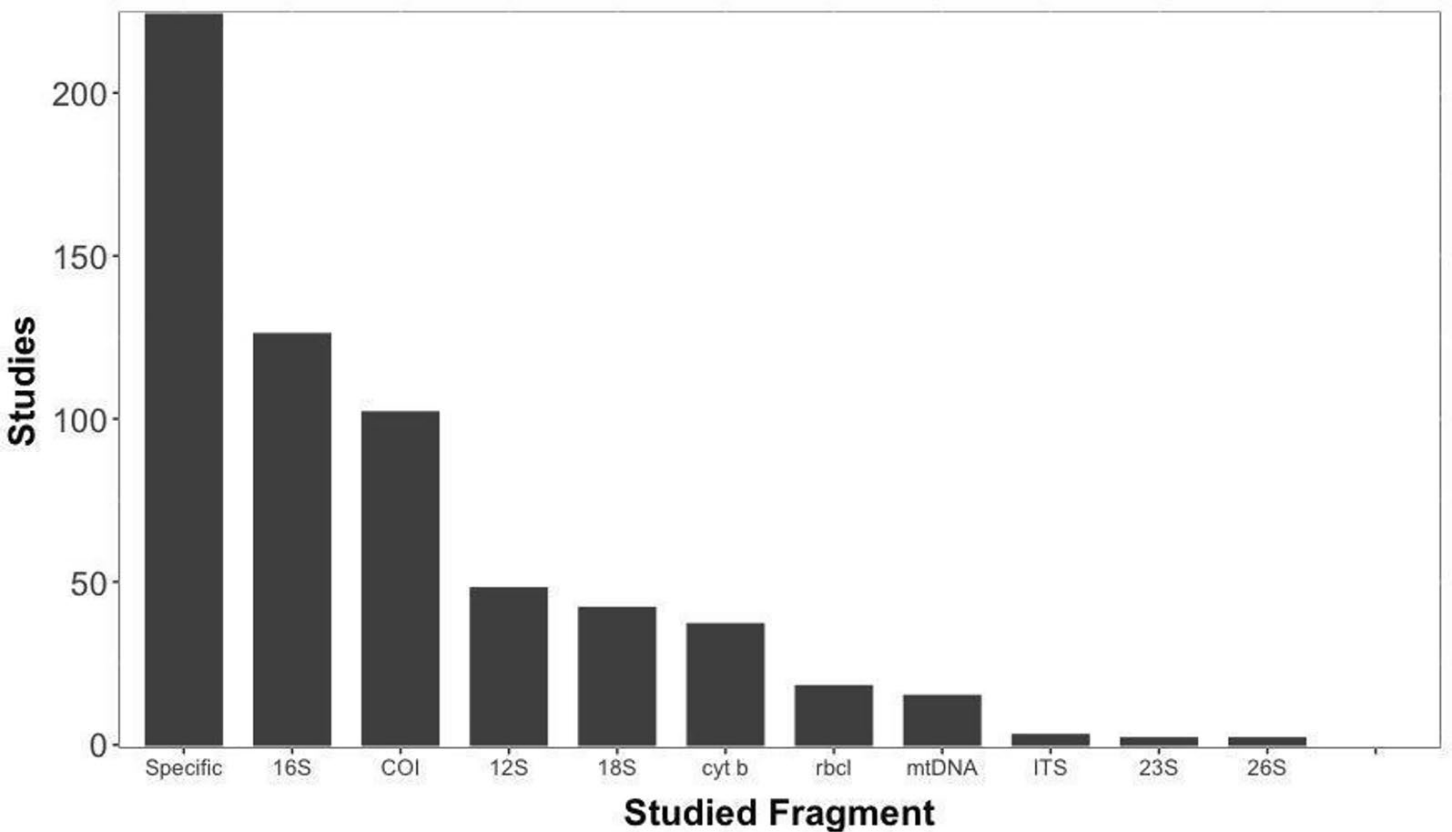
Number of studies for the reported conserved fragments. “Specific” fragments are studies where a specific primer for a single species or a set of species in the study were used to analyze an eDNA sample

The results for conserved fragments indicate that 41% or 225 studies did not use a conserved fragment for taxonomy (Fig. 4), and instead used a specific primer for the species they were interested in. 16S and COI were the most commonly used conserved fragment (126 and 102 studies respectively), while the least commonly used were the 26S and 23S fragments (only 2 studies each). Hypervariable regions were reported in only 10% of studies. The V4 and V3 regions were the most used hypervariable regions, with 36 and 21 studies respectively. The least used was the V2 region, which was only used in 2 studies (Additional file 13).

### Principal techniques used for eDNA processing, sequencing platforms employed, and availability of data access

Regarding sequencing techniques, we found that the most used is amplicon (462), followed by WGS (53) and only 4 studies that used transcriptomics (Table 4). The most common sequencing platform is Illumina (198), especially from 2012 onward (Table 4). However, most studies did not report a sequencing platform (290 or 53%). Finally, 229 studies (42%) did not provide access to sequencing data (runs, metadata, etc.).

**Table 4:** Number of studies per year, and the techniques approach, and sequencing platforms

Year	Technique				Platform							
	Amplicon	WGS	Transcriptomics	Not Specified	454 Roche s. 2005	Applied biosystems s. 2007	Illumina s. 2007	Ion Torrent s. 2010	Life technologies s. 2007	Nanopore s. 2014	Takara s. 2003	Not specified
2003	2		0	0	NA	NA	NA	NA	NA	NA	0	2
2004	0	0	0	1	NA	NA	NA	NA	NA	NA	0	1
2005	1	0	0	0	NA	NA	NA	NA	NA	NA	0	1
2006	0	0	0	0	0	NA	NA	NA	NA	NA	0	0
2007	0	0	0	0	0	0	0	NA	0	NA	0	0
2008	1	0	0	0	0	1	0	NA	0	NA	0	0
2009	3	0	0	0	0	0	0	NA	0	NA	0	3
2010	3	0	1	0	0	2	0	0	0	NA	0	2
2011	7	0	0	0	1	2	0	0	0	NA	0	4
2012	8	0	0	1	0	0	1	0	0	NA	1	7
2013	11	0	0	1	1	2	1	0	0	NA	0	8
2014	9	2	0	5	1	1	1	0	0	0	0	13
2015	20	3	2	1	1	0	7	1	1	0	0	16
2016	36	1	0	1	1	2	7	2	0	0	0	24
2017	44	2	0	2	3	0	12	3	0	0	0	30
2018	60	6	0	2	0	1	22	1	5	0	0	40
2019	57	8	0	2	1	3	24	2	0	0	0	40
2020	75	9	0	8	0	2	42	1	3	0	0	44
2021	92	10	1	2	0	2	55	1	1	2	1	43
2022	33	12	0	0	0	2	31	0	1	0	0	9
Total	462	53	4	26	9	20	203	11	11	2	2	287

### Type of sample and environmental matrix

The review showed that the most used environmental matrix for eDNA extraction in rivers is water with 451 studies, followed by mixed samples (i.e., containing sediment and water) (38), sediment (40), and biofilms (18) (Additional file 14).

### Thematic synthesis

**Fig. 5:**
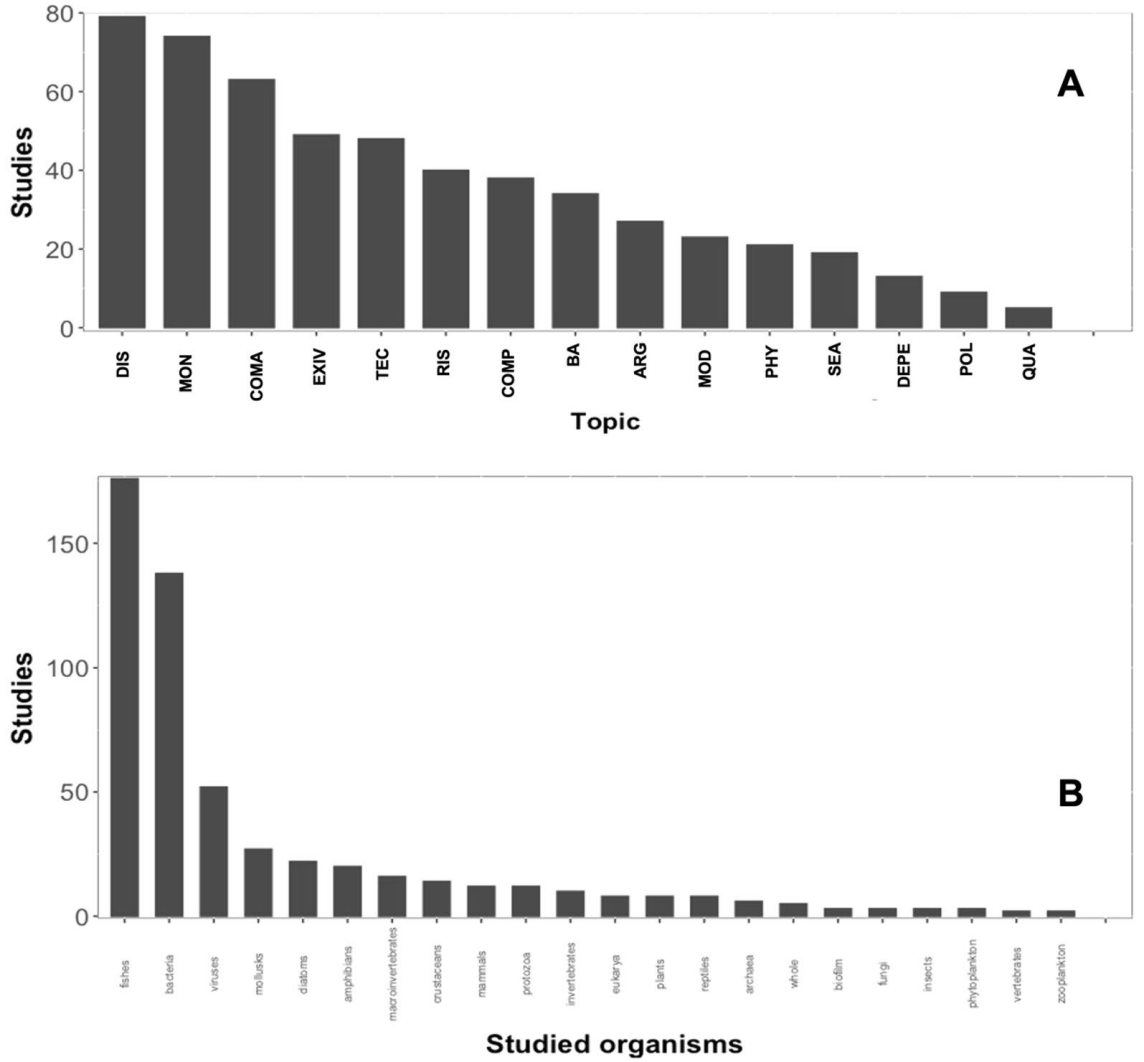
(**A**) Number of studies corresponding to each identified topic of interest for the thematic synthesis. (**B**) Number of papers per taxonomic group

The main goals of implementing eDNA in rivers can be synthesized in the 15 categories of applications and questions that can be answered using the eDNA framework (Table 2). The topic with the greatest number of published papers is Diseases Approach (DIS), followed by Monitoring (MON) and Community Assembly (COMA) with 79, 74, and 63 studies, respectively. The themes with fewer studies were Degradation/Perdurance (DEPE) and Quality Assessments (QUA) with 9 and 5 studies, respectively (Fig. 5A). The taxonomic group with most studies are fishes (176 or 32%), followed by bacteria (138) and viruses (52). Fungi were the least studied group with only 3 studies (Fig. 5B). However, some studies assessed more than one taxonomic group (e.g. fishes and amphibians), characterized all possible organisms regardless of their taxonomic classification ("whole" category in the results), or used other classification terms ("phytoplankton"). Fishes are the most studied group, because they are more conspicuous organisms, usually have more economic importance and their biology could facilitate the proof of the surveys done with eDNA. Also, bacteria and viruses are important for public health issues.

### Limitations of the map

Although some missing terms in the search string might have modified the results, we included a broad range of terms and refined the search with several trials among the search team, as can be stated by the consistency check of the key papers; therefore, we consider this bias to be low. We suggest that the modification of some eligibility criteria such as language, book chapters, meta-analysis could limit the rejection of some positive papers, because some of the studies launched that did not pass the inclusion criteria were mainly by the language limitation. However, as the aim of the mapping study was to provide an analysis of the trends in the most relevant work, the analysis of more than 500 papers can provide a great insight into the general trends.

Only English terms were included in the search strings since the main journals in the field are published in English and due to limitations of linguistic capabilities, also, gray literature in most cases, can only be accessed via institutional request. However, there is conflicting evidence on whether language restrictions increase the risk of language bias [44]. Another limitation is the actual terminology on eDNA, as in some papers the term EoDNA is used as a synomym of eDNA [45].

eDNA metabarcoding is highly sensitive to cryptic species and more cost-efficient than morphology-based studies [2, 6, 22, 23, 25–30]. It can be used to explore diversity beyond taxon identifications, and to characterize entire communities, but this mapping has shown that most studies analyze only a small set of focal taxonomic groups or species. For example, many studies use fishes as indicator species, and are not taking advantage that many more taxonomic groups used as indicator species (e.g. macroinvertebrates) could be analyzed in parallel from the same sample. This optimization of the use of the obtained sample could facilitate the implementation of biomonitoring programs.

## Conclusions

### Implication for policy/management

This review shows that eDNA has been used to investigate species composition or biological communities in rivers but access to this data is still limited. The results show that the majority of publications related to eDNA are focused on fishes, bacteria, and viruses. The most commonly used genetic markers are 16S and Cytochrome C oxidase Subunit I (COI). Ribosomal RNAs (rRNAs), specifically 12S, 16S, and 18S, are also frequently used for DNA metabarcoding [46, 47].

One of the main results of this review is that there is a gap in eDNA information in middle- and low-income countries, possibly indicating that costs and technology demand (equipment, installations, materials and institutions that support studies of this type) could be hindering its wider implementation. Despite the claims in the literature that NGS has become a key technology with supposedly lower costs than traditional species detection and identification [48, 49]. This can be seen clearly in the case of America, where the United States is the country with the most studies globally, but Latin America (except for Brazil) has a very low coverage of eDNA information.

The limited use of eDNA in the Global South could be due to: a) technical problems in practice (such as standardization, availability of databases, and the technology to cover multiple taxa); b) costs of eDNA survey, c) human resources (trained in taking the water samples, laboratory and field abilities, bioinformatic formation) and d) the support of the method by the society via science communication [46]. Another reason for a limited use of eDNA in these countries could be attributed to the climatic conditions that tend to show higher temperatures and humidity since these factors modify the lifetime of the sample [45, 46].

However, the results of eDNA must be treated with some reservation, considering the potential error, generated by false positives or negatives, the lack of genomic repositories where genomic data could be compared and the bias or potential errors related to sampling; that is why it should be ideal that this data could be corroborated and complemented with conventional survey methods. Also, the engagement of policymakers, stakeholders, and decision makers through the use of eDNA, will help to channel and design resources and efforts to characterize the communities’ composition, identify invasive, endangered or important commercial species, as well as determine the integrity of the freshwater systems, which provides a plethora of ecosystem services.

It is worth to mention that for the purpose of this study the open access for data, was considered as studies that included a link to the databases generated, that provided the SRA, Bioproject or Accession numbers to genomic sequence, or a link to Additional file 15. Studies that put data available on request were excluded because not all people can request adequately the necessary information, or considering the time that can be spent to reach this information.

### Implication for research

The clear bias of studies realized in developed countries shows that there a need to foster these types of technologies and make their potential advantages to assess ecosystems in such a way that a better decision making on biodiversity conservation will be possible.

We propose that areas of opportunity that must be addressed to ensure a better and robust implementation of the eDNA use for studying rivers, are the: (a) analyzing broader taxonomic groups, (b) establishing river sections to be studied (upper, mid or lower) and determine differences and timing, (c) a creation of a general free access database and datasets for biological collections and regions used to identify species; and (d) there is a limited number of studies that try to characterize the quality or ecological integrity of a river system, as well as the effects of pollution in these characteristics of a river, as can be seen in the reduced number of studies in categories QUA (Quality) and POL (Pollution).The main gaps are (i) a lack of a standardized methodology and genomic regions employed for taxonomic groups. (ii) missing information of the spatial changes in availability, transport, degradation of genomic material and zonation of eDNA in the different sections of moving waters (upper, mid, and lower), so including a multidisciplinary focus employing geomorphological, remote sensors, drones, and optical sensor will greatly enhance the understanding of processes about eDNA in moving waters.

This study presents a comprehensive analysis of the available evidence regarding the implementation of the eDNA methods in rivers. The findings indicate that since the year 2003, this approach has been applied to aquatic lotic systems, and their recent increase can be attributed to the development of Next-Generation-Sequencing technologies and their reduced costs. However, there is a bias towards high-income countries, particularly USA and Europe. Widespread use and applications of this approach at a global level would allow for the generation of a large amount of information that can be compared between countries to understand if responses of aquatic systems follow similar patterns worldwide; primarily, because this approach is crucial for comprehending aspects related to groups that cannot be studied using conventional methods, such as microorganisms, or that may be underestimated (cryptic species) due to morphological similarities within certain species groups. This enables a more realistic assessment of the biodiversity in tropical rivers, which are renowned as significant reservoirs of biodiversity.

Finally, it was asserted that eDNA is an approach with a positive cost–benefit ratio in relation to conventional methods for studying rivers. However, this assertion does not hold universally, as despite the reduced costs associated with sample collection and processing, it remains a costly and complex method, especially in countries lacking the necessary infrastructure to implement such a technique (laboratories, field materials, trained personnel for equipment use, etc.). Typically, these countries belong to the Global South. Therefore, if this limitation is not swiftly and appropriately addressed in such countries, the gap in the implementation of this approach, and consequently, the generated information, could potentially widen instead of narrowing.

## Supplementary Information

**Additional file 1:** Simplified conceptual model about eDNA use in rivers assessments.

**Additional file 2: **Simplified conceptual model for this systematic mapping.

**Additional file 3: **Consecutive methodologies used for systematic mapping and thematic synthesis.

**Additional file 4:** Link to PROCEED PROTOCOL “PROCEED-22-00006”.

**Additional file 5: **Word strings used in each of the searches and platforms.

**Additional file 6: **List of Key papers used in the Comprehensiveness of the search.

**Additional file 7: **Decision tree for screening stages.

**Additional file 8: **Search results exported from WoS, SCOPUS and Google Scholar.

**Additional file 9: **Studies used for the Kappa value calculation for 3 raters.

**Additional file 10: **Metadata sheet format used in the present study.

**Additional file 11: **Database with the studies that fullfilled inclusion criteria in all screening stages and their extracted data.

**Additional file 12: **Countries that have used the eDNA framework in rivers, and the number of studies launched by the searches.

**Additional file 13: **Hypervariable regions reported in the studies.

**Additional file 14: **Number of studies using different environmental matrix.

**Additional file 15: **ROSES form for systematic map protocol.

## Data Availability

Watch Additional material.
