# Supplementary material for: Existing evidence on the use of environmental DNA as an operational method for studying rivers: a systematic map and thematic synthesis
Source: Environ Evid. 2024 Feb 15;13:2. doi: 10.1186/s13750-024-00325-6 (PMC11376102; doi:10.1186/s13750-024-00325-6)
Supplement: Supplementary file 1 — Additional file 1: Simplified conceptual model about eDNA use in rivers assessments. [file 13750_2024_325_MOESM1_ESM.docx]

Read Me

Simplified conceptual model about eDNA use in rivers assessments. (Supp Material 1)

October 2023

Cruz-Cano et al.


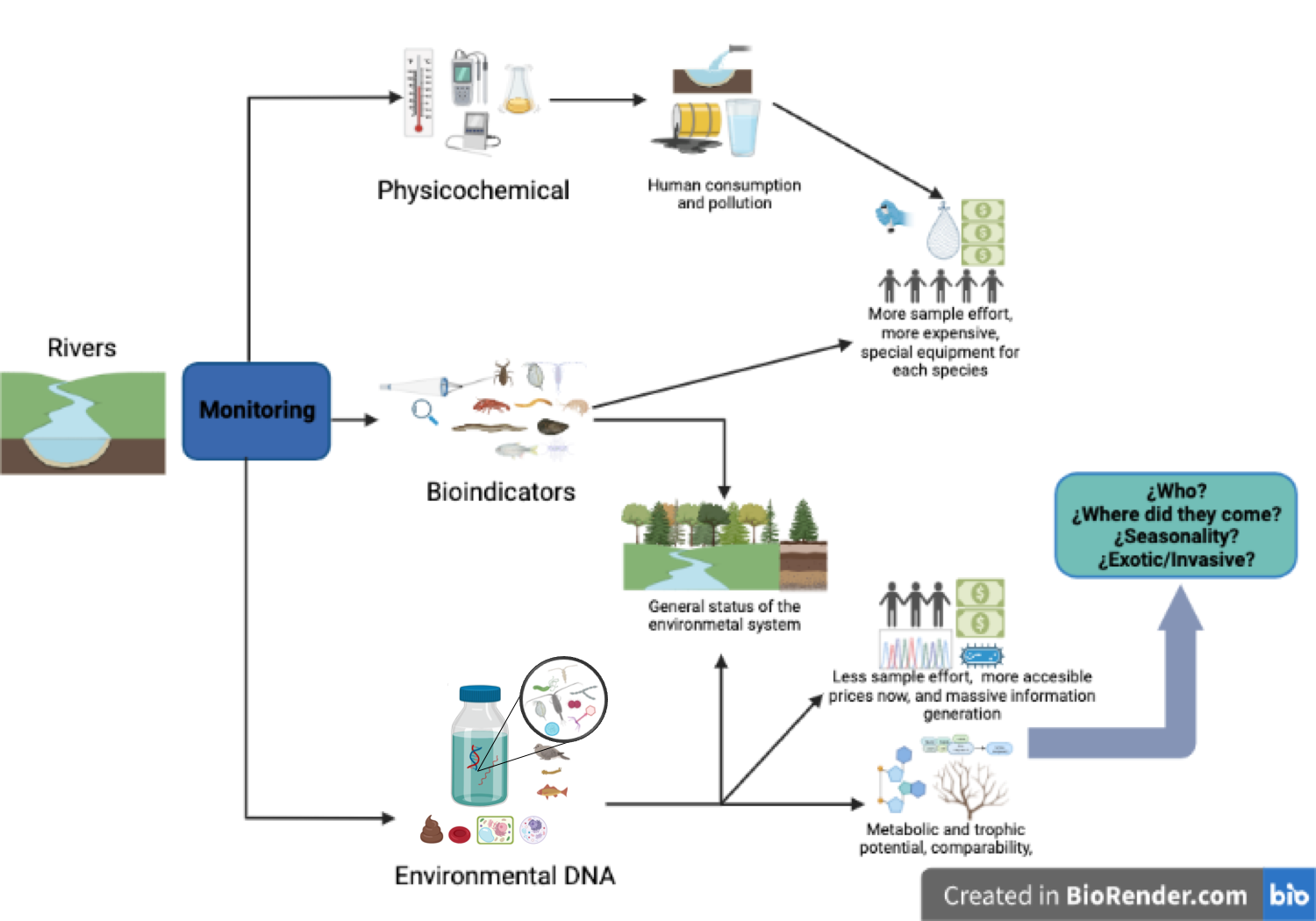


Although physicochemical and biomonitoring have been widely used globally for ecological and human-consume approaches, this monitoring type requires a higher sample effort, special equipment, taxonomic expertise and are expensive. In the other hand, environmental DNA is a technique that can cover multiple taxa, it can be “homogenizated” to different rivers type, and the quantity and resolution of information provided allows the generation of complementary information beyond only taxonomic information.
