# Supplementary material for: Existing evidence on the use of environmental DNA as an operational method for studying rivers: a systematic map and thematic synthesis
Source: Environ Evid. 2024 Feb 15;13:2. doi: 10.1186/s13750-024-00325-6 (PMC11376102; doi:10.1186/s13750-024-00325-6)
Supplement: Supplementary file 2 — Additional file 2: Simplified conceptual model for this systematic mapping. [file 13750_2024_325_MOESM2_ESM.docx]

Read Me

Simplified conceptual model for this systematic mapping (Supp Material 2)

October 2023

Cruz-Cano et al.


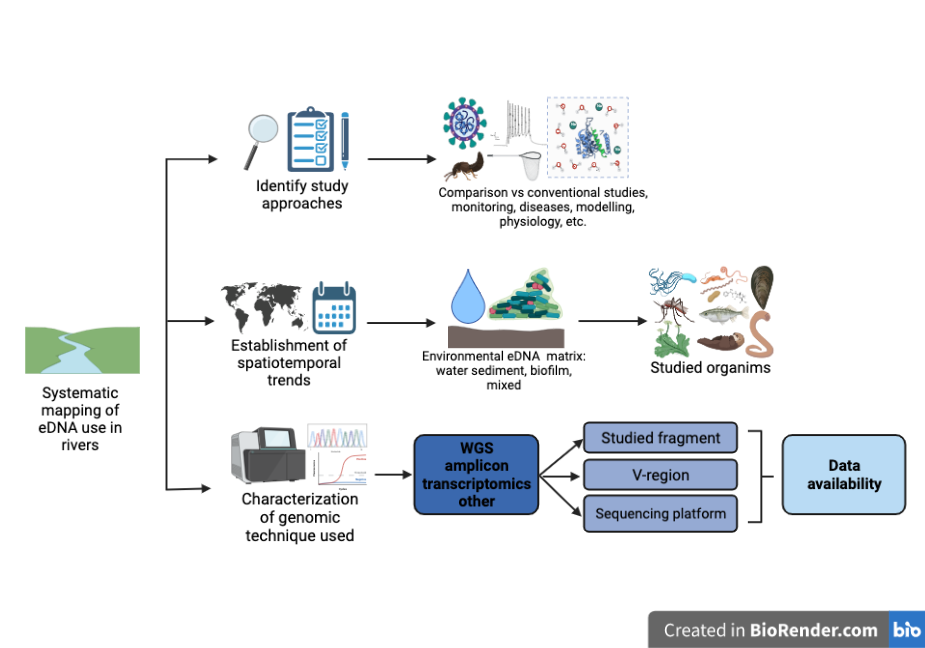


The study of rivers through the eDNA approach has been recently applied. However, there is a lack of evidence of how this approach is applied globally, and what kind of information generated is the predominant. In this study, the following information in the figure will be identified and extracted in order to identify global trends about eDNA use in rivers.
