# Supplementary material for: Existing evidence on the use of environmental DNA as an operational method for studying rivers: a systematic map and thematic synthesis
Source: Environ Evid. 2024 Feb 15;13:2. doi: 10.1186/s13750-024-00325-6 (PMC11376102; doi:10.1186/s13750-024-00325-6)

Read Me

Consecutive methodologies used for bibliometric analysis, systematic mapping and thematic synthesis (Supp Material 3).

Note: Bibliometric Analysis was made as a prospective and previous step to our systematic mapping, and thematic synthesis

October 2023

Cruz-Cano et al.


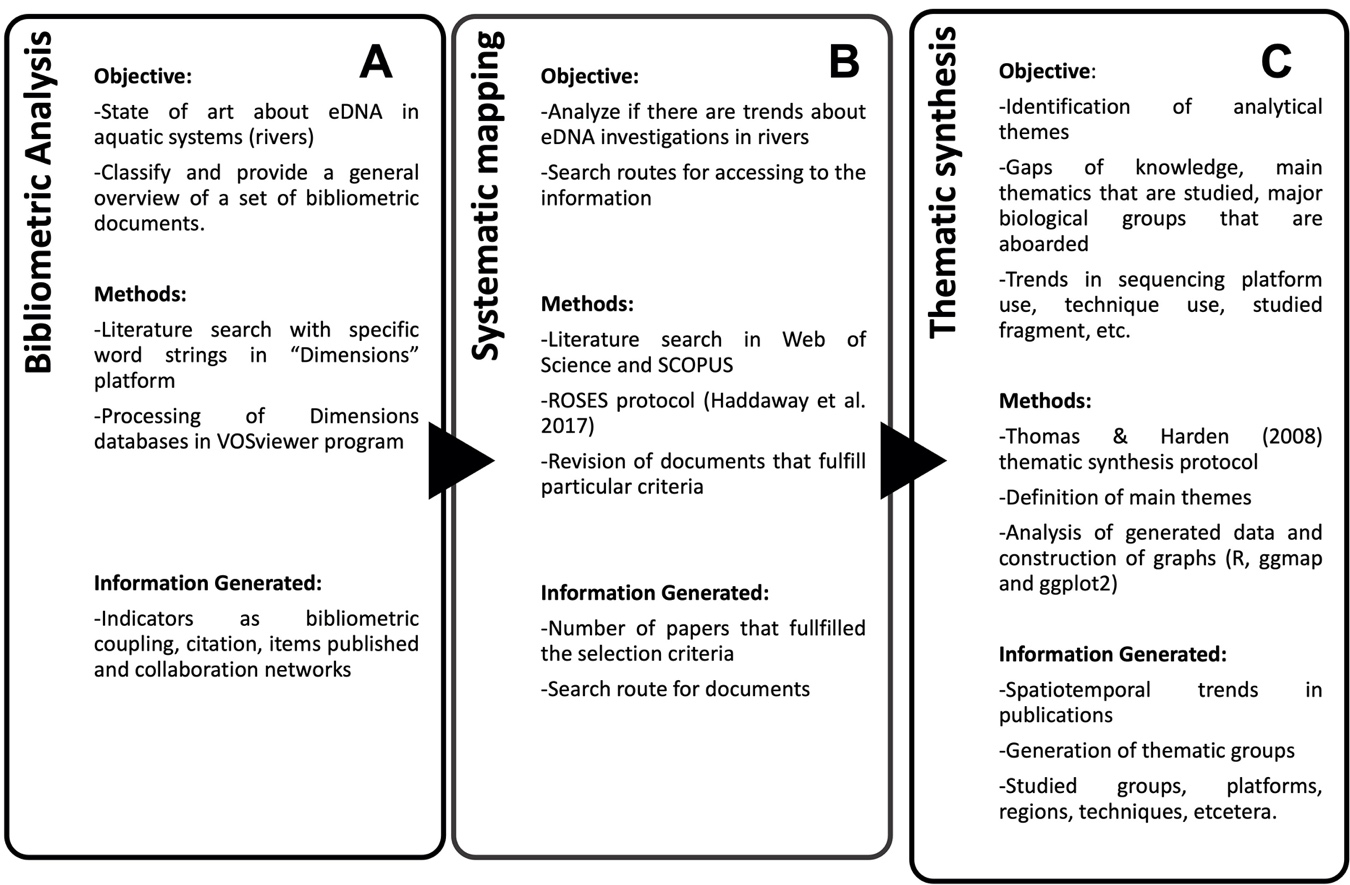

Supplement: Supplementary file 3 — Additional file 3: Consecutive methodologies used for systematic mapping and thematic synthesis. [file 13750_2024_325_MOESM3_ESM.docx]
