# Supplementary material for: Existing evidence on the use of environmental DNA as an operational method for studying rivers: a systematic map and thematic synthesis
Source: Environ Evid. 2024 Feb 15;13:2. doi: 10.1186/s13750-024-00325-6 (PMC11376102; doi:10.1186/s13750-024-00325-6)
Supplement: Supplementary file 4 — Additional file 4: Link to PROCEED PROTOCOL “PROCEED-22-00006”. [file 13750_2024_325_MOESM4_ESM.docx]

Read Me

Link to PROCEED PROTOCOL PROCEED-22-00006 (Supp Material 4)

October 2023

Cruz-Cano et al.

<https://www.proceedevidence.info/protocol/view-result?id=6>
