# Supplementary material for: Existing evidence on the use of environmental DNA as an operational method for studying rivers: a systematic map and thematic synthesis
Source: Environ Evid. 2024 Feb 15;13:2. doi: 10.1186/s13750-024-00325-6 (PMC11376102; doi:10.1186/s13750-024-00325-6)
Supplement: Supplementary file 5 — Additional file 5: Word strings used in each of the searches and platforms. [file 13750_2024_325_MOESM5_ESM.docx]

Read Me

Word strings used in each of the searches and platforms (Supp Material 5)

October 2023

Cruz-Cano et al.

The searches listed in the supplemental material were performed with different combinations of the terms referred to the river.

WoS: ((((TS=(environmental DNA)) OR TS=(eDNA)) OR TS=(genom*)) OR TS=(metagenom*)) AND TS=(river) and Article (Document Types).

SCOPUS:   TITLE-ABS-KEY ( {environmental DNA} OR edna ) AND ( river* OR stream OR lotic OR watershed OR catchment OR basin OR riparian OR watercourse OR waterway OR brook OR tributary OR channel OR creek )

Scholar:   (environmental DNA OR edna OR genom* OR metagenom*) AND (river* OR stream OR lotic OR watershed OR catchment OR basin OR riparian OR watercourse OR waterway OR brook OR tributary OR channel OR creek) "TITLE ABS KEY"

| **Number** | **Search term** | **Web of Science** | **Google_Scholar** | **Scopus** |
| --- | --- | --- | --- | --- |
| 1 | river | ALL=(environmental DNA OR eDNA OR genome OR metagenome) AND ALL=("river") | allintitle: (environmental DNA OR eDNA OR genome OR metagenome) ("river") | TITLE(environmental DNA OR eDNA OR genome OR metagenome) TITLE("river") |
| 2 | rivers | ALL=(environmental DNA OR eDNA OR genome OR metagenome) AND ALL=("rivers") | allintitle: (environmental DNA OR eDNA OR genome OR metagenome) ("rivers") | TITLE(environmental DNA OR eDNA OR genome OR metagenome) TITLE("rivers") |
| 3 | riverine | ALL=(environmental DNA OR eDNA OR genome OR metagenome) AND ALL=("riverine") | allintitle: (environmental DNA OR eDNA OR genome OR metagenome) ("riverine") | TITLE(environmental DNA OR eDNA OR genome OR metagenome) TITLE("riverine") |
| 4 | stream | ALL=(environmental DNA OR eDNA OR genome OR metagenome) AND ALL=("stream") | allintitle: (environmental DNA OR eDNA OR genome OR metagenome) ("stream") | TITLE(environmental DNA OR eDNA OR genome OR metagenome) TITLE("stream") |
| 5 | streams | ALL=(environmental DNA OR eDNA OR genome OR metagenome) AND ALL=("streams") | allintitle: (environmental DNA OR eDNA OR genome OR metagenome) ("streams") | TITLE(environmental DNA OR eDNA OR genome OR metagenome) TITLE("streams") |
| 6 | lotic | ALL=(environmental DNA OR eDNA OR genome OR metagenome) AND ALL=("lotic") | allintitle: (environmental DNA OR eDNA OR genome OR metagenome) ("lotic") | TITLE(environmental DNA OR eDNA OR genome OR metagenome) TITLE("lotic") |
| 7 | watershed | ALL=(environmental DNA OR eDNA OR genome OR metagenome) AND ALL=("watershed") | allintitle: (environmental DNA OR eDNA OR genome OR metagenome) ("watershed") | TITLE(environmental DNA OR eDNA OR genome OR metagenome) TITLE("watershed") |
| 8 | catchment | TI=(environmental DNA OR eDNA OR genome OR metagenome) AND ALL=("catchment") | allintitle: (environmental DNA OR eDNA OR genome OR metagenome) ("catchment) | TITLE(environmental DNA OR eDNA OR genome OR metagenome) TITLE("catchment") |
| 9 | basin | TI=(environmental DNA OR eDNA OR genome OR metagenome) AND ALL=("basin") | allintitle: (environmental DNA OR eDNA OR genome OR metagenome) ("basin") | TITLE(environmental DNA OR eDNA OR genome OR metagenome) TITLE("basin") |
| 10 | riparian | TI=(environmental DNA OR eDNA OR genome OR metagenome) AND ALL=("riparian") | allintitle: (environmental DNA OR eDNA OR genome OR metagenome) ("riparian") | TITLE(environmental DNA OR eDNA OR genome OR metagenome) TITLE("riparian") |
| 11 | watercourse | TI=(environmental DNA OR eDNA OR genome OR metagenome) AND ALL=("watercourse") | allintitle: (environmental DNA OR eDNA OR genome OR metagenome) ("watercourse") | TITLE(environmental DNA OR eDNA OR genome OR metagenome) TITLE("watercourse") |
| 12 | waterway | TI=(environmental DNA OR eDNA OR genome OR metagenome) AND ALL=("waterway") | allintitle:(environmental DNA OR eDNA OR genome OR metagenome) ("waterway") | TITLE(environmental DNA OR eDNA OR genome OR metagenome) TITLE("waterway") |
| 13 | brook | TI=(environmental DNA OR eDNA OR genome OR metagenome) AND ALL=("brook") | allintitle: (environmental DNA OR eDNA OR genome OR metagenome) ("brook") | TITLE(environmental DNA OR eDNA OR genome OR metagenome) TITLE("brook") |
| 14 | tributary | TI=(environmental DNA OR eDNA OR genome OR metagenome) AND ALL=("tributary") | allintitle: (environmental DNA OR eDNA OR genome OR metagenome) ("tributary") | TITLE(environmental DNA OR eDNA OR genome OR metagenome) TITLE("tributary") |
| 15 | channel | TI=(environmental DNA OR eDNA OR genome OR metagenome) AND ALL=("channel") | allintitle: (environmental DNA OR eDNA OR genome OR metagenome) ("channel") | TITLE(environmental DNA OR eDNA OR genome OR metagenome) TITLE("channel") |
| 16 | creek | TI=(environmental DNA OR eDNA OR genome OR metagenome) AND ALL=("creek") | allintitle: (environmental DNA OR eDNA OR genome OR metagenome) ("creek") | TITLE(environmental DNA OR eDNA OR genome OR metagenome) TITLE("creek") |
| 17 | rill | TI=(environmental DNA OR eDNA OR genome OR metagenome) AND ALL=("rill") | allintitle: (environmental DNA OR eDNA OR genome OR metagenome) ("rill") | TITLE(environmental DNA OR eDNA OR genome OR metagenome)TITLE("rill") |
