# Supplementary material for: Existing evidence on the use of environmental DNA as an operational method for studying rivers: a systematic map and thematic synthesis
Source: Environ Evid. 2024 Feb 15;13:2. doi: 10.1186/s13750-024-00325-6 (PMC11376102; doi:10.1186/s13750-024-00325-6)
Supplement: Supplementary file 6 — Additional file 6: List of Key papers used in the Comprehensiveness of the search. [file 13750_2024_325_MOESM6_ESM.docx]

Read Me

List of Key papers used in the Comprehensiveness of the search (Supp Material 6)

October 2023

Cruz-Cano et al.

1. Blabolil, P., Harper, L., Ricanova, S., Sellers, G., Muri, C., Juza, T., Vasek, M., Sajdlova, Z., Rychtecky, P., Znachor, P., Hejzlar, J., Peterka, J., Hanfling, B. 2021. Environmental DNA metabarcoding uncovers environmental correlates of fish communities in spatially heterogeneous freshwater habitats. *Ecological Indicators*. 126: 107698. <https://doi.org/10.1016/j.ecolind.2021.107698>.
2. Doi, H., Inui, R., Matsuoka, S., Akamatsu, Y., Goto, M., Kono, T. 2021. Estimation of biodiversity metrics by environmental DNA metabarcoding compared with visual and capture surveys of river fish communities. *Freshwater Biology*. 66: 1257-1266. DOI: 10.1111/fwb.13714
3. Mächler E., Salyani A., Walser J.-C., Larsen A., Schaefli B., Altermatt F., Ceperley N. 2021. Environmental DNA simultaneously informs hydrological and biodiversity characterization of an alpine catchment. *Hydrology and Earth Systems Sciences*. 25: 735-753. <https://doi.org/10.5194/hess-25-735-2021>.
4. Xie, R., Zhao, G., Yang, J., Wang, Z., Xu, Y., Zhang, X., Wang, Z. 2021. eDNA metabarcoding revealed differential structures of aquatic communities in a dynamic freshwater ecosystem shaped by habitat heterogeneity. *Environmental Research*. 201: 111602. <https://doi.org/10.1016/j.envres.2021.111602>.
5. Carraro, L., Mächler, E., Wuethrich, R., Altermatt, F. 2020. Environmental DNA allows upscaling spatial patterns of biodiversity in freshwater ecosystems. *Nature Communications*. 11:3585. <https://doi.org/10.1038/s41467-020-17337-8>.
6. Mächler, E., Salyani, A., Walser, J., Larsen, A., Schaefli, B., Altermatt, F., Ceperley, N. 2019. Water tracing with environmental DNA in a high-Alpine catchment. *Hydrology and Earth Systems Sciences.* <https://doi.org/10.5194/hess-2019-551>.
7. Li Y., Evans N.T., Renshaw M.A., Jerde C.L., Olds B.P., Shogren A.J., Deiner K., Lodge D.M., Lamberti G.A., Pfrender M.E. 2018. Estimating fish alpha- And beta-diversity along a small stream with environmental DNA metabarcoding. *Metabarcoding and Metagenomics*. 2:1-11. <https://doi.org/10.3897/mbmg.2.24262>.
8. Li, F., Peng, Y., Fang, W., Altermatt, F., Xie, Y., Yang, J., Zhang, X. Application of Environmental DNA Metabarcoding for Predicting Anthropogenic Pollution in Rivers. 2018. *Environmental Science & Technology*. 52: 11708-11719. <http://dx.doi.org/10.1021/acs.est.8b03869>.
9. Zimmermann, J., Gloeckner, G., Jahn, R., Enke, N., Gemeinholzer, B. 2015. Metabarcoding vs. morphological identification to assess diatom diversity in environmental studies. *Molecular Ecology Resources*. 15: 526-542. doi: 10.1111/1755-0998.12336
10. Mächler, E., Deiner, K., Steinmann, P., Altermatt, F. 2014. Utility of environmental DNA for monitoring rare and indicator macroinvertebrate species. *Freshwater Science*. 33: 1174-1183.
