# Supplementary figures and images for: Existing evidence on the use of environmental DNA as an operational method for studying rivers: a systematic map and thematic synthesis
Source: Environ Evid. 2024 Feb 15;13:2. doi: 10.1186/s13750-024-00325-6 (PMC11376102; doi:10.1186/s13750-024-00325-6)

Read Me

Decision tree for screening stages (Supp Material 7)

October 2023

Cruz-Cano et al.


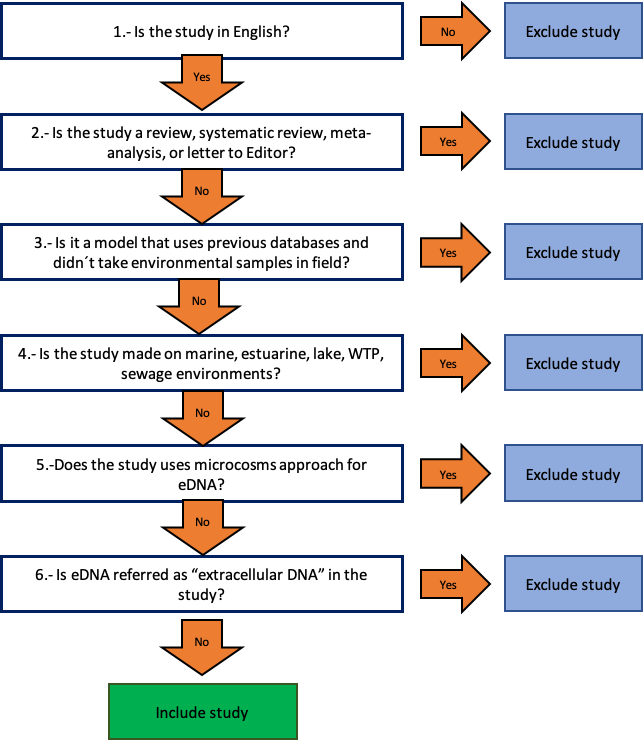

Supplement: Supplementary file 7 — Additional file 7: Decision tree for screening stages. [file 13750_2024_325_MOESM7_ESM.docx]

Read Me

Hypervariable regions reported in the studies. (Supp Material 15)

October 2022

Cruz-Cano et al.


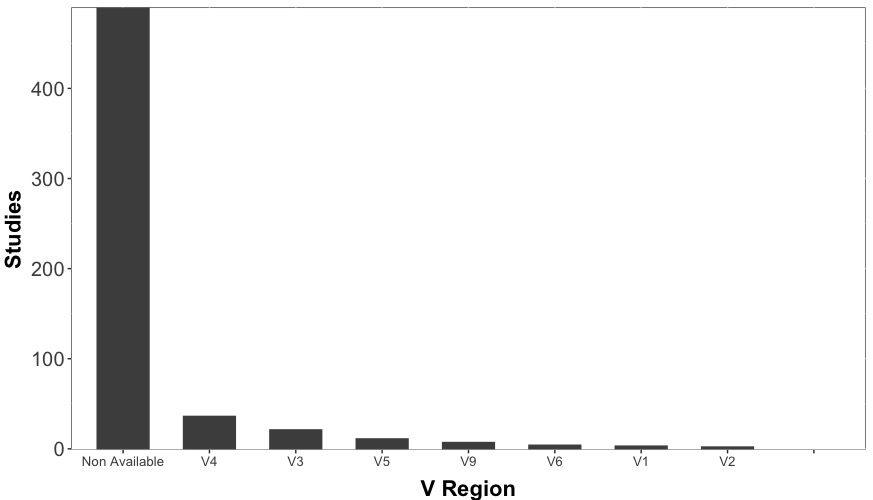

Supplement: Supplementary file 13 — Additional file 13: Hypervariable regions reported in the studies. [file 13750_2024_325_MOESM13_ESM.docx]
