# Supplementary material for: Existing evidence on the use of environmental DNA as an operational method for studying rivers: a systematic map and thematic synthesis
Source: Environ Evid. 2024 Feb 15;13:2. doi: 10.1186/s13750-024-00325-6 (PMC11376102; doi:10.1186/s13750-024-00325-6)

Read Me

Number of studies using different environmental matrix (Supp Material 16)

October 2022

Cruz-Cano et al.


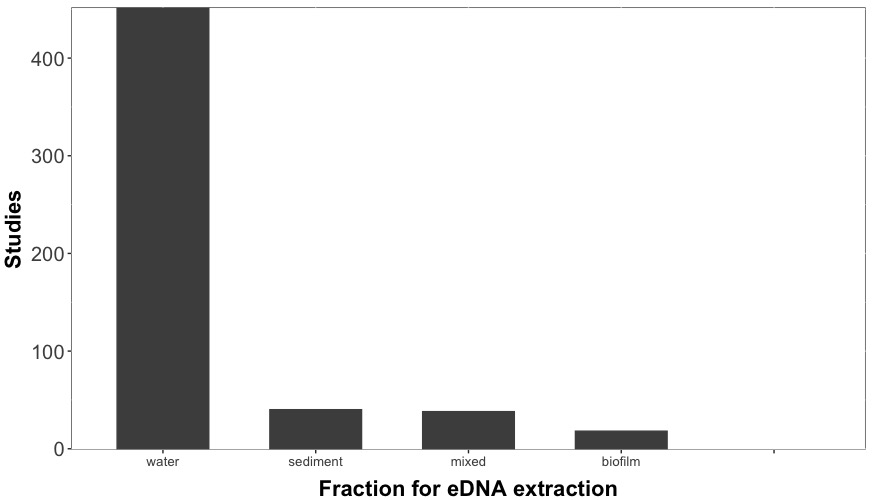

Supplement: Supplementary file 14 — Additional file 14: Number of studies using different environmental matrix. [file 13750_2024_325_MOESM14_ESM.docx]
